# Intensive blood pressure lowering: a practical review

**DOI:** 10.1186/s40885-020-00153-z

**Published:** 2020-11-01

**Authors:** Miguel Camafort, Josep Redón, Wook Bum Pyun, Antonio Coca

**Affiliations:** 1 Department of Internal Medicine-ICMiD. Hospital Clínic, University of Barcelona, Villarroel 170, 08036, Barcelona, Spain; 2 Cardiovascular Risk, Nutrition and Aging Research Group. IDIBAPS, Barcelona, Spain; 3 Ciber-OBN, Instituto de Salud Carlos III, Madrid, Spain; 4 Hypertension Clinic. Hospital Clinico, University of Valencia, Valencia, Spain; 5 Department of Cardiology, Ewha Womans University. Seoul Hospital, Seoul, South Korea

**Keywords:** Intensive blood pressure lowering, Ageing, Obesity, cardiovascular risk, Diabetes mellitus type 2, Ischemic heart disease, Chronic kidney disease, Heart failure, Ambulatory blood pressure measurement

## Abstract

According to the last Hypertension guideline recommendations, it may be concluded that intensive BP lowering is only advisable in a subgroup of patients where there is a clear net benefit of targeting to lower BP goals. However, taking into account the relevance of correct BP measurement, estimates of the benefits versus the harm should be based on reliable office BP measurements and home BP measurements.

There is still debate about which BP goals are optimal in reducing morbidity and mortality in uncomplicated hypertensives and in those with associated comorbidities. In recent years, trials and meta-analyses have assessed intensive BP lowering, with some success. However, a careful examination of the results shows that current data are not easily applicable to the general hypertensive population.

This article reviews the evidence on and controversies about intensive BP lowering in general and in specific clinical situations, and the importance of obtaining reliable BP readings in patients with hypertension and comorbidities.

## Background

Recently, we have experienced a change, in Hypertension Guidelines recommendations, to a more intensive lowering of BP levels. These changes are based on some trials that are far from including different hypertensive populations and therefore should be only applied to this populations. On the other hand, intensive BP lowering should be applied only when there is a clear balance in favour of benefits against adverse effects or harm caused by intensive BP lowering.

This article reviews the evidence on and controversies about intensive BP lowering in general and in specific clinical situations, and the importance of obtaining reliable BP readings in patients with hypertension and comorbidities.

## How low should we go?

Hypertension (HT) is defined as non-optimal levels of measured blood pressure (BP). More specifically, the ESH/ESC 2018 Guidelines on Hypertension, defines HT as the level of BP at which the benefits of treatment unequivocally outweigh the risks of treatment, as documented by clinical trials [1].

The importance of HT is increasing daily due to its high prevalence. For example, in 2010, it was estimated that one third of the total adult population worldwide was hypertensive; interestingly the percentage in high, low and middle-income countries was similar [2]. Currently, it is estimated that HT affects at least a billion adults.

High BP is known to be associated with a higher prevalence of cardiovascular disease (CVD), chronic kidney disease (CKD) and cognitive impairment and is, therefore, a major risk factor for cardiovascular death and disability. Prospective observational studies have shown a continuous, strong, positive, and independent relationship between BP levels and CVD [3, 4]. This applies to systolic BP (SBP) and diastolic BP (DBP). Likewise, pooled evidence from prospective cohort studies suggests that the minimum risk BP level for CVD could be a SBP of 110 mmHg to 115 mm Hg [5, 6].

Therefore, HT management guidelines have carefully established when to begin BP lowering treatment, and the BP goal to be achieved by treatment. The guidelines also describe the BP goal levels to which BP has to be reduced in special situations such as old and very old patients, and patients with diabetes and/or CKD. This is very important, since the balance between the potential benefits and potential harm or adverse effects due to a specific BP lowering goal must be considered.

The ESC/ESH 2018 Hypertension management guidelines [1] strongly suggested that lowering office SBP to < 140 mmHg is beneficial for all patient groups, including independent older patients, and that there is evidence to support targeting SBP to 130 mmHg for most patients, if tolerated. Even lower SBP levels (< 130 mmHg) may potentially be beneficial for some patients, especially to further reduce the risk of stroke, if well tolerated. SBP should not be targeted at < 120 mmHg because the balance of benefit versus harm becomes concerning at these levels.

The 2017 ACC/AHA Hypertension Guidelines recommended a BP target of < 130/80 mmHg in adults with known CVD or moderate-to high CVD risk. A reasonable target for subjects with no additional marker of increased CVD risk should also be < 130/80 mmHg [7]. A further recommendation is that some patients will benefit from an SBP target of < 120 mmHg, especially those at high risk of CVD. However, considering the clinical applicability of this last recommendation, in-depth analysis of the available evidence shows that the specific inclusion and exclusion criteria of any RCT related to this particular question may limit extrapolation to a more general population with hypertension. In addition, BP measurements in all the relevant trials were more consistent with guideline recommendations than is common in clinical practice, resulting in lower absolute SBP values.

Nevertheless, BP lowering is almost always referred to SBP, but when it comes to intensive BP lowering, it would be interesting to know how far SBP and DBP are controlled, as both components have different pathophysiology and risk factors and which component of BP (or both) is hindering from comprehensive management. Among treated hypertensive. In an analysis from the KNHANES, Cho SMJ and cols [8] have shown that in treated hypertensive patients SBP control rates were 77.5%, DBP control rates were 87.4%, and S&DBP control rates were 71.6%. They also found that highest SBP control rate in those treated for hypertension (96.2%) was found on those aged 30–39 years, and that DBP control rate progressively increased with older age, with lower DBP control rate was observed in men with lower education level, higher household income, and heavy drinkers. We should not forget that lifestyle changes have also different effect on the different components of BP, being more effective on SBP [9].

In Asia, the 2018 Korean Hypertension Guidelines [10] recommend intensive BP lowering in all risk patients including patients with clinical cardiovascular, cerebrovascular or renal disease [11], and the elderly, but the guidelines also recommend taking into account the specificities of diseases associated with HT, the method of BP measurement and the lack of indubitable evidence in some fields.After the publication of the KSH guidelines Kwun SJ [12] and cols evaluate the potential impact of the 2018 KSH guidelines on hypertension management status among the Korean population in terms of prevalence of hypertension, antihypertensive medical treatment recommendations, and control status in Korean adults. They conclude that a more intensive BP lowering goa would result in a remarkable increase in the number of adults who are recommended to receive medical treatment, and a decline in the hypertension control rate. This study suggests that there is a large scope for improvement in BP control in Korean adults.

Therefore, according to guideline recommendations, it may be concluded that intensive BP lowering is only advisable in a subgroup of patients where there is a clear net benefit of targeting to lower BP goals. However, taking into account the relevance of correct BP measurement, estimates of the benefits versus the harm should be based on reliable office BP measurements and home BP measurements.

## Intensive BP lowering and older people

The benefits and adverse effects of intensive BP lowering was the main goal of a meta-analysis, which included 4 trials involving 10,857 older hypertensive patients [13]. Assessment of the efficacy and safety of intensive BP-lowering strategies in hypertensive patients aged more than 65 years, intensive BP lowering was associated with reduction of 29% in major cardiovascular events (MACE), 33% in cardiovascular mortality, and 37% in heart failure (HF) compared with standard BP lowering. Rates of myocardial infarction (MI) and stroke did not differ between the two groups and there was no significant difference in the incidence of serious adverse events. However, as patients in the intensive BP lowering arm had a higher number of antihypertensive medications and, as older patients, very frequent comorbidities, there was always a possibility of an increased incidence of adverse events. In addition, a BP goal where the benefits were superior to adverse events or more events could not be established, as 3 of the 4 trials achieved a mean SBP of 136 mmHg in the intensive BP lowering arm and the other 123 mmHg.

It remains unclear whether intensive BP lowering is well-tolerated and if its effects are uniform across the age spectrum. Byrne [14] analyzed the efficacy and safety across the age spectrum in the SPRINT trial and found no differences by age, whether tested continuously or categorically (*P* > 0.05). Nevertheless, the authors of the Sprint Trial recognized the possibility of bias in the trial design and development, as clinicians were not blinded to the randomized assignment. Likewise, during follow-up, participants in the intensive arm were seen for unscheduled clinic visits about 20 to 30% more often than those in the standard arm [15].

When talking about BP lowering in older patients, frailty must always be considered. Williamson et al. [16] in an exploratory subgroup analysis from the SPRINT trial stratified by baseline frailty, found higher event rates with increasing frailty, although there were significantly lower event rates in the intensive treatment group. The same results were found by stratifying by gait speed in favour of the intensive treatment group. However, the definition of frailty depends on the tool used for its measurement. In the SPRINT trial, the Rockwood approach [17] and gait speed were used. In a recent analysis, Russo [18] assessed the degree of frailty in SPRINT and found that in this sub-study all institutionalized patients were excluded, which may have affected the degree of frailty. They concluded that the SPRINT results can only be applied to the general population but not to “frail” older patients. Other relevant questions could be automatic BP measuring in the absence of the attending physician and the greater use of diuretics. Therefore, further studies are required to define a safe BP target, and these should be tailored specifically to different degrees of frailty and pre-frailty.

## Is intensive BP lowering effective and safe in patients with a high body mass index?

It is unclear whether intensive BP management is well-tolerated and affects risk uniformly across the body mass index (BMI) spectrum. In a post-hoc analysis of the SPRINT trial using restricted cubic splines, Oxlund at al [19] investigated the relationship between BMI, response to intensive BP lowering, and clinical outcomes. The results showed that intensive BP lowering consistently had the same efficacy and safety across the BMI spectrum of patients included in SPRINT, and therefore may represent an important cardiovascular risk reduction strategy in obese hypertensive patients.

## Intensive BP lowering according to total CV risk

Another aspect is whether intensive BP reduction by treatment has differential outcome effects in patients with different baseline risk scores, particularly when searching for a balance between safety and efficacy. In a post-hoc analysis of the SPRINT trial, Zhang etal [20] categorized participants into low-risk, intermediate-risk, or high-risk arms, according to the Framingham risk score (FRS). Intensive BP control was beneficial throughout the three risk categories and was similar in patients with different FRS. However, the benefits were accompanied by a higher risk of serious adverse events.

## Intensive BP lowering in diabetic hypertensive patients

It remains unclear whether intensive BP lowering is beneficial for diabetic hypertensives and whether this strategy would be influenced by baseline BP or CVD risk. In a post-hoc analysis, Rahman et al. [21] found no evidence of differences in the beneficial BP lowering effects, regardless of baseline SBP (even below 120 mmHg; P for heterogeneity, 0.85), DBP (even below < 70 mmHg; *P* = 0.49), or when the 10-year CVD risk was ≥20% or < 20% (*P* = 0.08). The effects of randomized treatment on treatment discontinuation due to cough or hypotension/ dizziness were also statistically consistent across subgroups defined by baseline BP and CVD risk (all *P* ≥ 0.08). The authors concluded that adults with diabetes mellitus appear to benefit from more intensive BP treatment even at levels of BP and CVD risk that some guidelines do not currently recommend. However, as this was a post-hoc analysis, a randomized controlled trial would be necessary to ensure whether hypertensive diabetic patients would benefit from a more intensive BP lowering therapy.

Wang et al. [22] aimed to clarify whether intensive BP lowering was associated with benefits in patients with type 2 diabetes mellitus. They included 16 RCTs in a meta-analysis comparing intensive vs. less intensive BP lowering and found that intensive BP lowering resulted in significant reductions in the all-cause mortality risk, MACE, MI, stroke, CV death, and the progression of albuminuria. Nevertheless, there was no uniformity between studies, with completely different inclusion criteria, and different intensive BP lowering goals, and therefore the results are not conclusive.

Another unanswered question is the effect of intensive BP lowering in hypertensive patients with coronary artery disease (CAD) and DM. In a sub-study of the HIJ-CREATE study, Kamishima et al. [23] found that the relationships between achieved BP and the incidence of MACE did not follow a J-shaped curve. Intensive SBP lowering to < 120 mmHg did not correlate with an increased risk of MACE. The authors suggested that intensive BP lowering may not impair patients’ clinical courses, even in a high-risk population.

Another interesting issue is the potential impact of intensive antihypertensive treatment on the risk of new onset diabetes. A pre-specified study by Roumie et al. [24] assessed whether intensive BP lowering lowered the risk of new onset diabetes mellitus in the SPRINT trial. There was a risk reduction in some risk groups, such as those with CKD. In contrast, intensive BP lowering was not associated with an increased risk for new diabetes mellitus but was associated with more impaired fasting glucose (IFG) (adjusted HR 1.17 (95% CI 1.06–1.30) *p* = 0.002). However, specific medications were not analysed as a cause of impaired glucose metabolism and studies are needed to assess whether any medications may be associated with fasting glucose impairment.

## Intensive BP lowering in chronic kidney disease and worsening renal function

CKD is associated with higher mortality and morbidity. Might intensive BP lowering have an effect on all-cause mortality? In a meta-analysis of 18 randomized clinical trials comprising 15,924 patients with CKD stages 3 to 5, Malhotra et al. [25] aimed to answer this question. The mean baseline SBP was 148 mmHg in both arms (more intensive and less intensive). Mean SBP fell by 16 mmHg in the more intensive treatment arm and by 8 mmHg in the less intensive arm. More intensive BP control resulted in a 14% lower risk of all-cause mortality. However, most patients had CKD stage 3, so the risks and benefits in more advanced CKD are not known. Another limitation is that baseline BP and the extent of BP reduction in the randomized treatment arms differed across the individual trials. Therefore, the meta-analysis cannot offer an optimal BP target in patients with CKD.

With respect to the efficacy of intensive BP lowering in CKD patients on CVD and renal outcomes, Cheung [25] analysed the SPRINT participants with and without CKD at baseline. For the primary composite cardiovascular outcome, the hazard ratio [HR] was 0.81 (95% CI; 0.63 to 1.05) in favour of intensive BP lowering. The intensive group also had a lower rate of all-cause death (HR, 0.72; 95% CI, 0.53 to 0.99). Treatment effects did not differ between participants with and without CKD (P for interaction 0.30). As for the pre-specified main kidney outcome (composite of ≥50% decrease in eGFR from baseline or ESRD), there was no difference between groups (HR, 0.90; 95% CI; 0.44 to 1.83). However, after 6 months the intensive group had a slightly higher rate of change in eGFR (20.47 versus 20.32 ml/min per 1.73 m^2^ per year; *P* = 0.03). Intensive SBP reduction was generally well tolerated by participants with CKD, although hypokalemia and hyperkalemia were more common in the intensive group, probably due to more frequent use of medications, such as diuretics and renin-angiotensin system inhibitors.

Another important question, namely the relationship between CKD and stroke and the influence of intensive BP lowering, was assessed by Agarwal et al. [26] in a post-hoc analysis of the Secondary Prevention of Small Sub-cortical Strokes (SPS3) Trial. They found that baseline CKD was significantly associated with an increased risk of recurrent stroke, but the effects of intensive lowering on SBP were not influenced by baseline CKD status. Again, conclusive evidence for this will require adequately powered studies in patients with moderate-to-advanced CKD.

A particular concern regarding intensive BP lowering is what reduction in renal function is acceptable? The initial decline is attributed to a reduction in the glomerular filtration rate but intensive BP lowering carries the risk of iatrogenic renal ischemia. The usual recommendation is to taper BP lowering if serum creatinine increases by more than 30% or if there is a lowering of eGFR of 20%. Collard et al. [27] assessed the relationship between changes in eGFR and BP and between the initial eGFR reduction and annual eGFR reduction during follow-up in a post hoc analysis of the SPRINT and ACCORD-BP trials. The authors found that a reduction of 26% in eGFR may be considered normal after a 10 mmHg reduction in mean BP and with an additional eGFR reduction of 3.4% for every 10 mmHg of mean BP reduction. In addition, the initial eGFR decline was not associated with a greater annual eGFR decline during a mean follow up of 3.2 years. However, the ACCORD-BP trial included patients with albuminuria and excluded those with serum creatinine ≥1.5 mg/dL, while the SPRINT trial included patients with CKD and eGFR > 20 ml/min. Therefore, in the post-hoc analysis, the percentage of patients with CKD was about 20%. The authors concluded that, although an initial eGFR reduction may be observed following intensive BP lowering, this is not associated with a persistent decline in renal function during follow up. Therefore, decisions on the tapering of BP-lowering therapy after an initial eGFR decline should be made taking multiple measurements of renal function and the BP reduction achieved into account.

Malhotra et al. [28], in a longitudinal subgroup analysis of participants with CKD (defined as eGFR < 60 ml/min/1, 73 m^2^) from the SPRINT trial, analysed the effect on eight urine biomarkers of tubule cell damage despite loss of eGFR and differences between groups. Despite a more pronounced lowering in eGFR in the intensive arm, none of the eight tubule marker levels were higher in the intensive arm compared with the standard arm. Two tubule function markers (B2M and A1M) were 29% (95% CI, 10 to 43%) and 24% (95% CI, 10 to 36%) lower at year 1 in the intensive versus the standard arm. Although the results are not applicable to persons with diabetes, and few participants had advanced CKD, the results seem to indicate that eGFR declined in the intensive arm of SPRINT predominantly due to hemodynamic changes rather than to intrinsic damage in kidney tubule cells and may, therefore, be considered as a pseudo worsening of renal function.

With respect to albuminuria, Xie et al. [29] conducted an updated systematic review and meta-analysis aimed at assessing the efficacy and safety of intensive BP-lowering strategies. In 19 trials including 44, 989 participants they found that more intensive BP-lowering (achieved mean BP levels of 133/76 mmHg) compared with less intensive BP-lowering (achieved mean BP levels of 140/81 mmHg) significantly reduced the risk of progression of albuminuria [RR reduction of 10%; 3–16%], defined as new onset micro-albuminuria/macro-albuminuria or a change from micro-albuminuria to macro-albuminuria).

## Intensive BP lowering and coronary heart disease

In a post-hoc analysis of the International Verapamil-Trandolapril Study (INVEST), Messerli et al. [30] determined whether low BP was associated with excess mortality and morbidity in 22,576 patients with hypertension and coronary heart disease (CHD) included in the trial. They found that the relationship between BP levels, after adjustment, followed a J-shaped relationship between DBP and the primary outcome (combination of all-cause death, nonfatal stroke, and nonfatal MI, all-cause death, total MI, and total stroke). The J-shaped curve was also present in the analysis of CHD as a secondary endpoint.

Subsequently, the SPRINT trial [31] showed that, in non-diabetic patients with a high risk of cardiovascular events, intensive BP lowering (goal SBP < 120 mmHg) compared with the standard SBP target of < 140 mmHg, resulted in lower rates of fatal and nonfatal MACE and death from any cause. But the results of the secondary outcomes showed that neither as for MI nor acute coronary syndrome, intensive BP lowering was associated to reductions in events.

## Intensive BP lowering and cerebrovascular disease

The SPS3 trial [32] was a secondary prevention trial that compared intensive and standard treatments for the reduction of recurrent stroke. The study clearly showed that the intensive treatment arm (mean SBP achieved 127 mmHg vs. 138 mmHg on standard treatment) significantly decreased the risk of cerebral bleeding by 63% but did not decrease the risk of the recurrence of lacunar infarcts.

Later, Katsanos et al. [33] conducted a systematic review and meta-regression analysis of the association between BP reduction and recurrent stroke and cardiovascular events using data from 14 randomized controlled clinical trials, including 42,736 patients, on secondary stroke prevention. Systolic BP reduction was linearly and significantly related with a lower risk of recurrent stroke, MI, death from any cause, and cardiovascular death. Similarly, DBP reduction was linearly related to a lower risk of recurrent stroke and all-cause mortality (*P* = 0.009). These results clearly show that strict and aggressive BP control toward normotension is essential for secondary stroke prevention.

## Intensive BP lowering and heart failure

Would intensive BP lowering lead to a greater lowering of the risk of left ventricular hypertrophy (LVH)? This was the question raised by Soliman et al. [20] in a new post-hoc analysis of the SPRINT trial. The results showed that in hypertensives without diabetes, intensive BP lowering resulted in lower rates of new LVH in those without LVH at baseline, and higher rates of LVH regression in those with existing LVH. In a meta-analysis of 9 RCT, Zhang et al. [34] explored the question further, assessing the effect of intensive BP lowering in incident heart failure. The pooled analysis of nine prospective, randomized controlled trials indicated that intensive SBP decrease the risk of HF in patients without diabetes and in those aged ≥65 years.

With respect to patients with established HF, a post-hoc analysis of the SPRINT trial by Upadhya et al. [35] assessed whether there a was differential reduction in acute decompensated heart failure (ADHF) events due to intensive BP treatment in the six key, pre-specified subgroups in SPRINT: age ≥ 75 years, prior CVD, CKD, women, black ethnicity, and three levels of baseline SBP (≤ 132 vs. > 132 to < 145 vs. ≥ 145 mm of Hg). The results showed that targeting an SBP of < 120 mmHg significantly reduced ADHF events and the benefit was similar across all key, pre-specified subgroups in comparison with SBP < 140 mmHg. Participants who developed ADHF had a markedly increased risk for subsequent CV events and death, highlighting the importance of strategies aimed at preventing ADHF, especially intensive BP reduction.

## Which measurement should we rely on?

Home BP and ambulatory BP values are very useful in predicting cardiovascular events independently of office BP. Nevertheless, there is an obvious lack of data on the most appropriate measurements in context of the most suitable way to measure intensive BP lowering.

The HONEST [36] (Home BP measurement with Olmesartan Naïve patients to Establish Standard Target BP) trial was an observational cohort study that found that morning home BP was an independent predictor for both ischemic heart disease and cerebrovascular disease, whereas office BP was not [20, 34]. The lowest CVD risk was observed at morning home systolic BP < 124 mmHg. Again, more data are needed before strong recommendations can be made.

Self-measured blood pressure at home (HBP) has commonly been used in clinical practice. Although unattended BP measurement (UBP), in which the patient is left alone before and during the measurement, has been investigated, the advantages of UBP over HBP or conventionally-measured office BP obtained using automated devices (CBP) remain unclear. Asayama et al. [37] performed a multicenter clinical study in Japan comparing unattended office BP (UBP), automated office BP (CBP), and HBP in 308 hypertensive patients. UBP and CBP were measured according to the SPRINT protocol. The authors found very low correlation coefficients for SBP when comparing UBP versus morning and evening HBP, whereas the correlation coefficient was 0.5 (*P* < 0.001) for DBP. The authors conclude that, based on the low correlations and the wide range of differences, UBP cannot be used as an alternative to HBP.

Höller et al. [38] conducted a single-centre study to evaluate the differences between office BP measurements methods, comparing CBP and UBP, auscultatory office BP (AOBP) and UBP and observed a significant difference between AOBP and UBP measurement for SBP and DBP. Likewise, Chrubasik et al. [39] compared different methods of measuring BP in 145 patients using ABPM, OBP, and HBP, and found limited agreement between the different methods of BP measurement.

## Summary and conclusions

In recent decades, the prevalence of HT has continued to increase globally, with only small improvements in HT control. HT remains the most common preventable risk factor for CVD, CKD and cognitive impairment, and the leading single contributor to all-cause mortality and disability worldwide.

There is still debate about which BP goals are optimal in reducing morbidity and mortality in uncomplicated hypertensives and in those with associated comorbidities. In recent years, trials and meta-analyses have assessed intensive BP lowering, with some success. However, a careful examination of the results shows that current data are not easily applicable to the general hypertensive population.

The only data from RCTs on diabetic hypertensive patients is the ACCORD trial, where patients with type 2 diabetes, at high risk for cardiovascular events, treated with antihypertensive drugs were randomized to an intensive vs. standard SBP goal. Intensive treatment did not reduce the rate of a composite outcome of fatal and nonfatal major cardiovascular events, and adverse events were significantly higher. Although some post-hoc analyses and meta-analyses of observational studies have been published since then, there is still no conclusive evidence on the optimal BP goal for these patients and, therefore, the recommendation of the ESH Guidelines to target SBP to 130 mmHg and < 130 mmHg if tolerated, but not < 120 mmHg, are not still definitive.

The number of CKD patients included in the SPRINT trial and the fact that most of them were in stage 3 should be considered when trying to apply intensive BP lowering to patients with advanced CKD. Common sense in clinical practice is to monitor creatinine, eGFR and urine albumin in these patients during BP lowering.

In older hypertensive patients, the most important aspect is frailty. To our knowledge, no RCT assessing intensive BP lowering in older people adjusted for a multidomain evaluation of frailty has been published. Until there are more robust data, it would be prudent to follow the current recommendations of the guidelines based on the best available evidence. Correct BP measurement, including home BP, a global geriatric evaluation and correct monitoring is highly recommended in these patients.

Heart failure continues to be an unknown territory for intensive BP lowering. Most patients are old and frail and there is very little data on ABPM measurement. With respect to heart failure with reduced ejection fraction, the fact that BP lowering treatment is modifying the disease makes it easier to manage, although it is not clear which BP goals are better and which treatment is more beneficial.

## Data Availability

Not applicable.
